# Supplementary material for: Chromosome-scale and haplotype-resolved genome assembly of a tetraploid potato cultivar
Source: Nat Genet. 2022 Mar 3;54(3):342–8. doi: 10.1038/s41588-022-01015-0 (PMC8920897; doi:10.1038/s41588-022-01015-0)
Supplement: Supplementary file 1 — Methods, Figs. 1–11 and refs. 1–13. [file 41588_2022_1015_MOESM1_ESM.pdf]

nature portfolio

https://doi.org/10.1038/s41588-022-01015-0

Supplementary information

Chromosome-scale and haplotype-resolved genome assembly of a tetraploid potato cultivar

In the format provided by the authors and unedited

Supplementary Information

Chromosome-scale and haplotype-resolved genome assembly of a tetraploid potato cultivar

Sun and Jiao *et al.*, 2021

Correspondence: Korbinian Schneeberger (schneeberger@mpipz.mpg.de)

Materials and Methods

HMW somatic DNA extraction, library preparation and sequencing (‘Otava’)

The genome of ‘Otava’ was sequenced with PacBio HiFi Sequel II platform with four SMRTcells. High-molecular-weight DNA was isolated from 1.5 gram material (Otava leaves) with a NucleoBond HMW DNA kit (Macherey Nagel). Quality was assessed with a FEMTOpulse device (Agilent) and quantity measured by fluorometry Quantus (Promega). A HiFi library was then prepared according to the manual "Procedure & Checklist - Preparing HiFi SMRTbell® Libraries using SMRTbell Express Template Prep Kit 2.0" with initial DNA fragmentation by g-Tubes (Covaris) and final library size binning by SageELF (Sage Science). Size distribution was again controlled by FEMTOpulse (Agilent). Size-selected libraries were sequenced on a Sequel II device at Max Planck Genome-centre Cologne (MP-GC) with Binding kit 2.0 and Sequel II Sequencing Kit 2.0 for 30 h. Details of read information are provided in Supplementary Table 1.

Pollen DNA extraction, library preparation and sequencing (‘Otava’)

Ten anthers from two ‘Otava’ flowers were extracted with forceps and submerged in woody pollen buffer (WPB). Around 800,000 pollen grains were extracted from the anthers by vortexing them in WPB. Isolated pollen was prefiltered (100 µm) and bursted (10 µm) using Celltrics™ sieves and WPB. After sorting and counting using the BD FACSAria™ Fusion flow cytometer with high-speed sort settings (70 µm nozzle and 70 PSI sheath pressure, at Max Planck Institute for Biology of Ageing), a total of 12,600 nuclei were selected and collected in a solution of 4.2 µL phosphate-buffered saline with 0.1% bovine serum albumin. According to manufacturer’s instructions, the nuclei were loaded into a 10x Genomics Chromium Controller in two batches each with 6,300 nuclei. This led to two 10x single cell CNV libraries (DNA), and both were sequenced with a Illumina HiSeq3000 device in 150 bp paired-end read mode at Max Planck Genome center (MPGC, Cologne, Germany), reaching a coverage of 52x per haplotype. Note, barcodes (in single cell sequencing) were corrected using *cellranger* (10x Genomics). Details of read information are provided in Supplementary Table 1.

Omni-C DNA extraction, library preparation and sequencing (‘Otava’)

An aliquot of HMW DNA was extracted from fresh leaves of ‘Otava’ used for a Dovetail Omni-C library created at MPGC using the Omni-C™ Kit. The library was sent to BGI, Hongkong (China) with dry ice, where it got sequenced on DNBSEQ-G400 platform. Details of read information are provided in Supplementary Table 1.

RNA library preparation and sequencing ('Otava')

RNA was isolated from leaves with an RNeasy plant kit, Qiagen including an on-column RNase treatment. Poly-A RNA was enriched from 1 µg total RNA by the NEBNext® Poly(A) mRNA Magnetic Isolation Module. RNAseq libraries were prepared as described in NEBNext Ultra™ II Directional RNA Library Prep Kit for Illumina (New England Biolabs). A total of eleven cycles were applied to enrich library concentration. Sequencing-by-synthesis was performed on a NextSeq 2000 with P3 chemistry and 2 x 150 bp read mode. Three replicates from three leaves of the same 'Otava' plant were made. Details of read information are provided in Supplementary Table 1.

Enzymatic methylome library preparation and sequencing ('Otava')

For Enzymatic Methyl-seq (EM-seq) genomic DNA was isolated with DNAeasy plant mini kit, Qiagen. Then 200 ng DNA was fragmented with COVARIS S2 to 300 bp including spke-ins as recommended by NEB. An Illumina-compatible library was prepared according to the NEBNext® Enzymatic Methyl-seq Kit protocol (NEB) with a total of 5 cycles to enrich barcoded library fragments. Sequencing-by-synthesis was performed on a NextSeq 2000 with P3 chemistry and 2 x 150 bp read mode. Three replicates from three leaves of the same 'Otava' plant were made. Note, the three leaves here were the same as those used for RNA extraction, where a leaf was cut into 2 pieces with one half for RNA sequencing and the other for methylation sequencing. Details of read information are provided in Supplementary Table 1.

DNA extraction, library preparation for linked-read sequencing

One 10x Genomics linked-read library (DNA load: 0.625 ng) was created using DNA extracted from leaves (NucleoBond HMW DNA kit, Macherey Nagel), after size-selection for ≥45 kb with a Sage Science BluePippin high-pass protocol (U1 marker, 0,75% cassette), respectively for 'Otava', 'Hera' and 'Stieglitz'[1,2]. The libraries were sequenced on Illumina HiSeq3000 platform in 150 bp paired-end read mode (at MPGC). The 'Otava' sequencing was used in sequencing depth analysis, while the parental sequencings were used in *k*-mer based haplotyping evaluation analysis. Details of read information are provided in Supplementary Table 1.

DNA extraction, library preparation and sequencing ('Stieglitz' and 'Hera')

Fresh leaves were sampled from the parental cultivar 'Hera', DNA was extracted using the Plant DNA Kit of Macherey-Nagel™, treated with RNAse, and an Illumina-compatible short read library was prepared after gDNA fragmentation (S2, Covaris) with an Ovation ultralow V2 library kit (Tecan Genomics). The library was sequenced using Illumina HiSeq3000 (at MPGC)

in 150 bp paired-end read mode. Similarly, an Illumina library was prepared and sequenced on the same device for the *Stieglitz* genome (NEBNext® Ultra™ II FS DNA Library Prep Kit for Illumina®). Details of read information are provided in Supplementary Table 1. Note that all parental genome data were used only for evaluating haplotyping accuracy in the haplotype-specific assemblies.

10x Genomics sequencing barcode correction

The improved version of the *DM* potato reference genome v4.04[3,4] was indexed with *cellranger-dna* (version 1.1.0, 10x Genomics), using sub-function *mkref* under default settings. For each of the two 10x single cell CNV libraries, reads were aligned to the *DM* genome using *cellranger-dna cnv* under default settings. The generated bam files were sorted with *samtools* with -*n* option, and reads updated with corrected barcodes were respectively extracted using customized code.

Selection of single-cell sequencings

The read sets of each of the single pollen genomes were aligned to the initial assembly of 6,366 contigs using *bowtie2*. After filtering out non-primary reads using *samtools view* with options "-*F* 3840 -*q* 1", read counts within each 50 kb coverage marker along each contigs were obtained using *bedtools* (v2.29.0)[5]. The coverage ratio of the assembly by a read set was calculated as the total size of covered coverage markers divided by the total assembly size (where a marker harboring more than $7*(N/10^6)$ reads was considered as covered, with N being the number of reads aligned to the assembly). Finally, 717 read sets each with over 70,000 read pairs (equivalent to 0.02x of the haploid genome size) which could cover the assembly with a ratio of 0.55 to 0.72 were selected to perform haplotype-specific contig grouping (Figure S4). Pooled alignments of these short reads to the initial assembly delivered solid coverage (above 17x) across 98.5% of the initial assembly, supporting that the 10x libraries had captured comprehensive parts of the genome.

Analysis of HiFi reads not assigned to any of the 48 haplotypes

The non-grouped reads were provided to *hifiasm* to perform independent assembly under default settings. All raw contigs were aligned to the NCBI nucleotide database using *blastn*[6] under default settings. The top blasting hit was selected to define whether a contig was from organellar genomes, and the percentage overlapping organelle genomes was calculated as the aligned length divided by the total contig length (Supplementary Figure 4).

Haplotype-specific Hi-C read separation

To avoid mis-joined haplotypes (which is a common problem in haplotyping and scaffolding with chromosome conformation capture data), we removed Hi-C read pairs linking different haplotypes. All Hi-C reads were first aligned to the initial assembly (of 6,366 contigs) using *bowtie2*. If a read pair can be aligned to coverage markers from a single group, then it was assigned to that group, otherwise the read pair was removed from analysis. Note that when a read pair could be aligned to coverage markers of multiple groups simutaneouly, it was randomly assigned to one of the groups. This process led to 70x per haplotype out of the raw coverage of 130x per haplotype.

Evaluation of assembly quality

The trimmed short reads from the two 10x single cell CNV libraries (DNA) were used to create a *k*-mer databse (*otava_genome.meryl*) for the 'Otava' genome using *meryl*[7] with options "*k=21 count threads=4 memory=8g*". Then the fully combined set of *Hera* 1,2 and *Stieglitz* 1,2 sequences (*otava_genome.fa*) were compared with the *k*-mer database (*otava_genome.meryl*) to investigate the completeness (97.3%) and the base accuracy (QV>51.7) of the final assembly, using "*merqury.sh genome.meryl otava_genome.fa full_genome*" (version 1.3)[7].

Identification of identical-by-decent blocks

The initial 50 kb coverage markers were aligned to the chromosome-level haplotype-resolved assembly using *minimap2*, where a maximum of 2,3,4 hits were allowed for diplotig, triplotig and tetraplotig related markers. Neighbouring (aligned) markers of the same type were connected into larger regions if their distance was less than 200 kb.

Identification of potentially collapsed variants within IBD blocks

All short reads of the two 10x single-cell CNV libraries were aligned to the polished full assembly using *bowtie2*. Bed files were created with coordinates of IBD blocks shared by two, three, and four haplotypes, and short reads aligned to these regions were extracted using *bedtools intersect.* For all groups of IBD blocks, a single-copy of the shared sequence from each group was collected, which was subsequently combined into one fasta file to create a set of sequences as reference, which was indexed by *bowtie2*. The extracted short reads were aligned to the created reference sequences using *bowtie2*. Variants were called using *bcftools*. The results were collected in Supplementary Table 7, where it showed a maximum of 1 SNP per 72 kb could have been missed from phasing in IBD blocks.

Characterization of noncoding RNA

First, all transcripts were assembled using *StringTie* (v1.3.4d) with default parameter settings using our RNA-seq data and public RNA-seq data. Transcripts overlapping with the exons of annotated protein-coding gene at the sense strand or shorter than 200 bp were removed with the script *FEELnc_filter.pl* in the tool *FEELnc*[8]. Second, the transcripts with a potential for protein-coding were filtered by the tool *CPC2*[9]. Third, we used the *blastx* to align the filtered transcripts against the protein sequences from Swiss-Prot database[10] to filter strong hits with some cutoffs (alignment identity > 35, alignment length >40 aa and alignment coverage of the query or subject sequence >35) following a previous study[11].

Gene family and PAV analysis

We clustered the protein sequences from all four haplotypes using *OrthoFinder* (version 2.2.6)[12] with default parameters. The gene presence/absence variants between the haplotypes were identified based on the gene groups resulting from *OrthoFinder*. To decrease the false absence of genes due to some residual missing genes in annotation, the focal haplotype was predicted to also contain a homolog of a gene annotated in other haplotypes when the gene was aligned to the focal haplotype with a high alignment coverage (>90%) and identity (>90%) and no loss-of-function variations (based on the variations calling from the tool *SyRI* as described above).

Crossover detection (at LG 4) in pollen genomes

For each of the four haplotype sequences at LG 4, 30x error-free Illumina paired-end reads were simulated using *pirs*[13] with options of *-m* 300 *-v* 10 *-l* 100 *-x* 25 *-e* 0 *-a* 0 *-g* 0. The reads were aligned to *Chr-4.Stie_2* as reference sequence using *bowtie2* under default settings, and variations were called using *bcftools* (version 1.9). For each of the non-reference sequences, we selected variations specific to all other haplotypes and with a minimum mapping quality of 150, minimum allele frequency of 0.99 and a coverage of the alternative allele in [16, 34]. For the reference chromosome, if all the other three haplotypes had the same allele but different from the reference, the allele would be selected as specific in the reference chromosome. Such variations were filterted for minimum mapping quality of 150, minimum allele frequency of 0.99 and a coverage of the alternative allele in [16, 34]. All these haplotype-specific variations were used as SNP markers in genotyping pollen genome sequencings.

Each read set of the 717 pollen nuclei sequencing was respectively aligned to the *Chr-4.Stie_2* sequence, and consensus were called using *bcftools*. The allele read counts were obtained at the above-defined SNP markers for chromosomes 4, based on which crossovers were detected.

Supplementary Figures

a

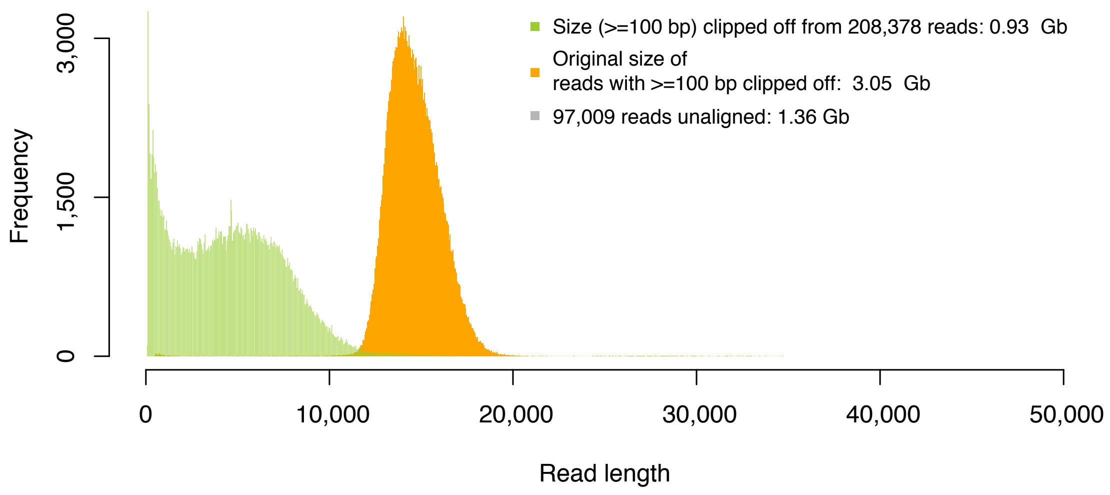

b

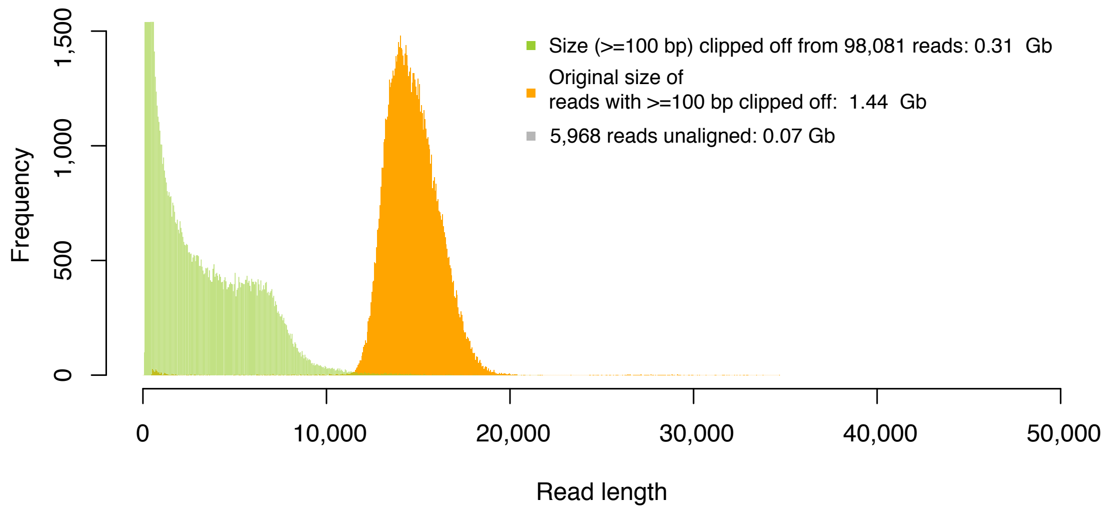

Supplementary Figure 1. Aligning (102.2 Gb) PacBio HiFi reads to the raw and the initial assemblies (after purging low-coverage contigs). a. Aligning HiFi reads to the raw assembly of 17,153 contigs led to 208,378 reads, which were clipped with >=100 bp (0.93 Gb bases clipped off) and 97,009 reads (1.36 Gb), which were not aligned. This gave an overall alignment rate of 97.8% at base level. **b.** Aligning HiFi reads to the (purged) initial assembly of 6,366 contigs (with an N50 of 2.1 Mb) led to 98,081 reads, which were clipped with >=100 bp (0.31 Gb bases clipped off) and 5,968 reads (0.07 Gb), which were not aligned. This gave an overall alignment rate of 99.6% at base level. The increased alignment rate supported that the purging had removed redundant information, for example, repeated representation of some genomic regions which could be induced by sequencing errors in HiFi reads, from the raw assembly. (*x*-axis in bp)

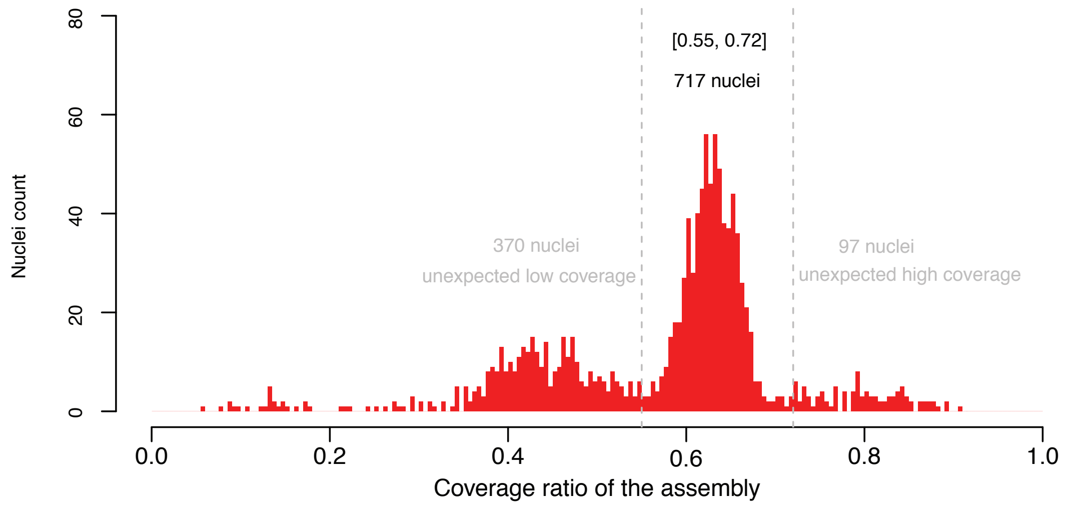

Supplementary Figure 2. Histogram of the coverage ratio of each pollen after aligning the respective reads against the initial assembly (of 6,366 contigs). Initially, 1,184 pollen nuclei (with a minimum of 40,000 151 bp read pairs) were extracted from the two 10x single cell CNV libraries. By aligning each read set to the initial assembly, we found a major cluster containing 717 nuclei that could cover the assembly with a ratio of 0.55 to 0.72 and selected them to perform linkage grouping, where the minimum read pair number was 70,356, overall leading to a mean coverage of 0.18x of the haploid genome.

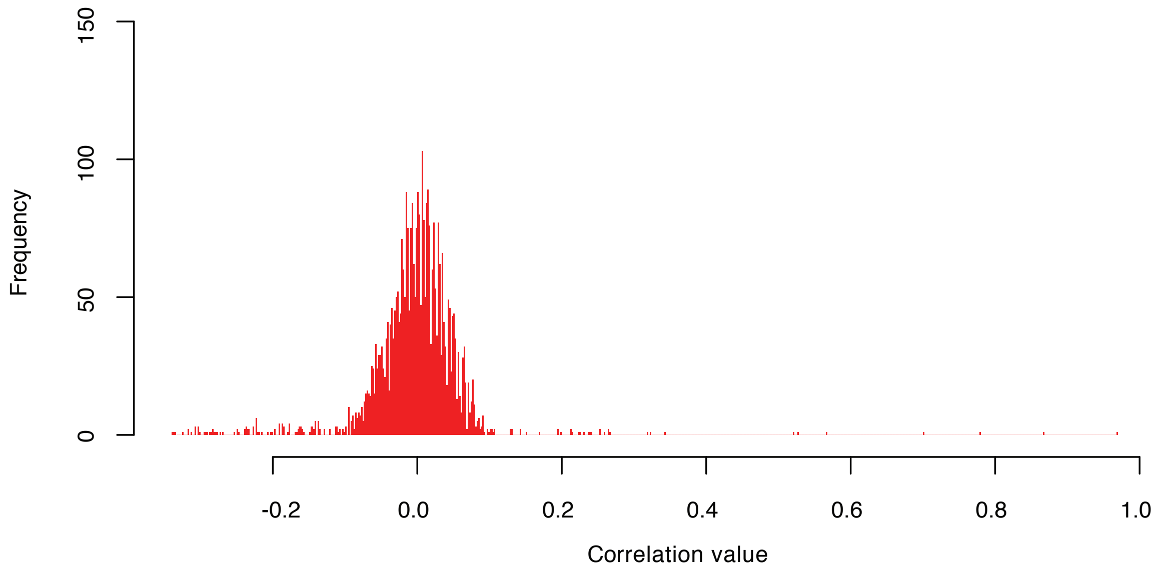

Supplementary Figure 3. Distribution of the PAP-defined correlation values of an arbitrarily selected representative coverage marker of one contig with the coverage markers of all other contigs. Based on this distribution, a minimum correlation value of 0.55 was selected as threshold to identify marker pairs, which were physically closely located. A maximum correlation value of -0.25 was selected to support that two markers could be from (neighboring) allelic regions on homologous chromosomes.

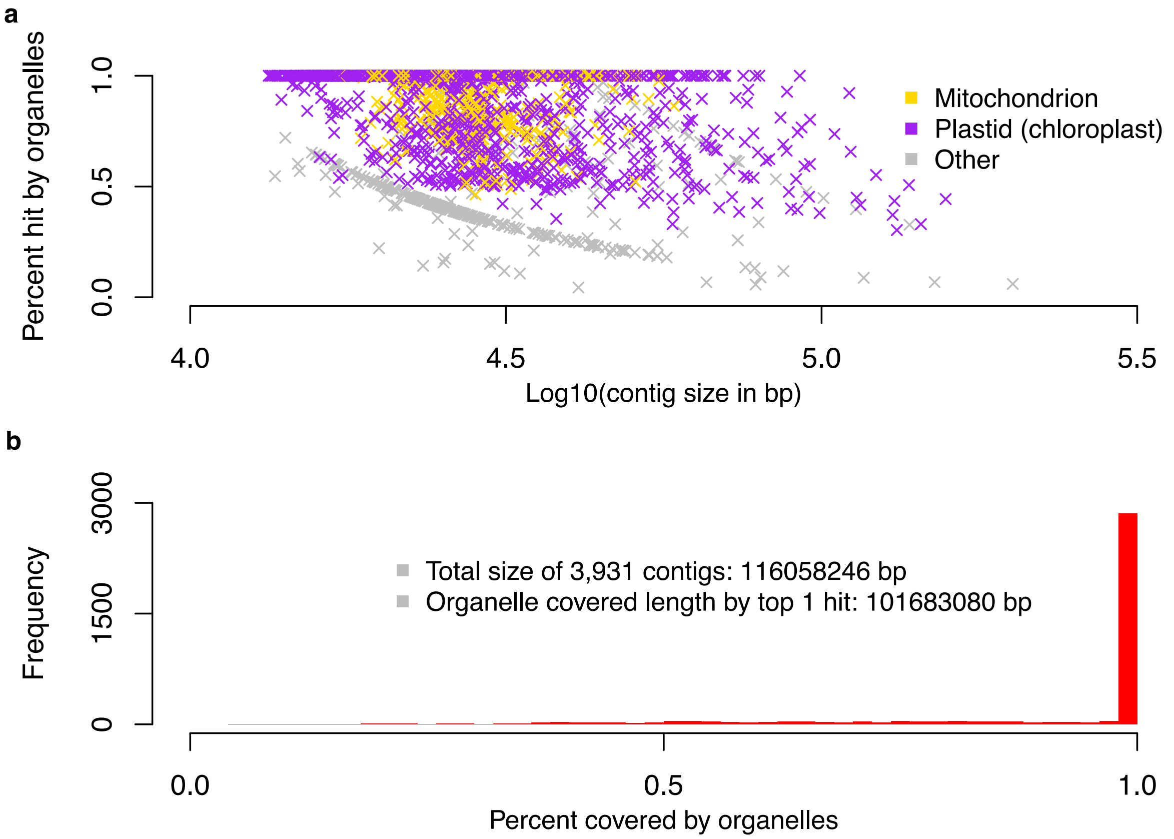

Supplementary Figure 4. Alignment of contigs (that were assembled from 9.9 Gb non-grouped HiFi reads) to the NCBI nucleotide database. Among all 3,931 contigs (with a total size of 116 Mb), 3,439 could be at least partially aligned to organelle sequences (**a**), among which 2,814 contigs fully overlapped organelle sequences (**b**). At base-level, 101.7 Mb sequences could be aligned to organelle genomes, accounting for nearly 88% of the total contig size of 116 Mb.

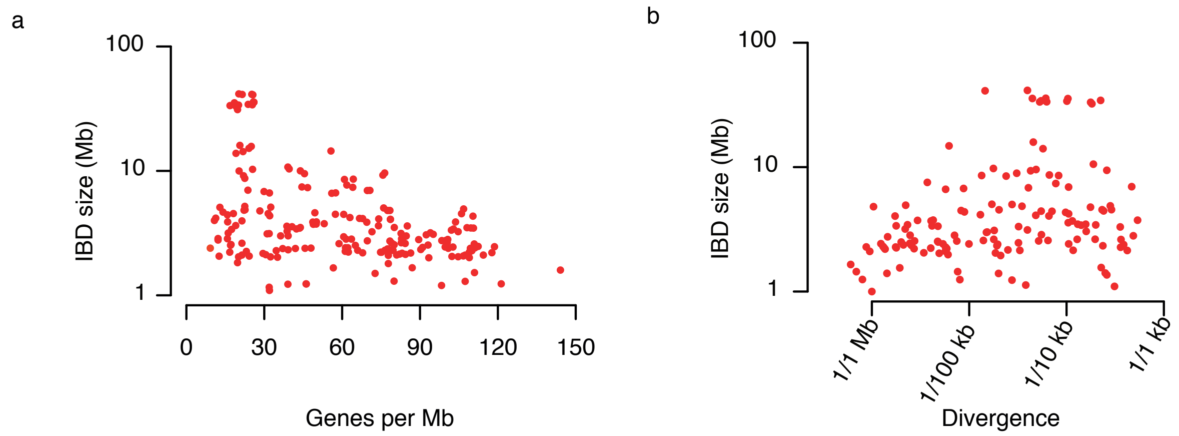

Supplementary Figure 5. Correlation of features at IBD blocks. a. Correlation of IBD block size with gene density. IBD blocks could reach over 40 Mb, however, such large IBD blocks located in peri-centromeric regions were usually with low gene density. b. Correlation of IBD block size with their age estimated based on the accumulated mutations. IBD blocks showed different levels of mutations indicating that larger IBD blocks were generally not younger than many of the smaller IBD blocks. Y-axis was in log10 scaled.

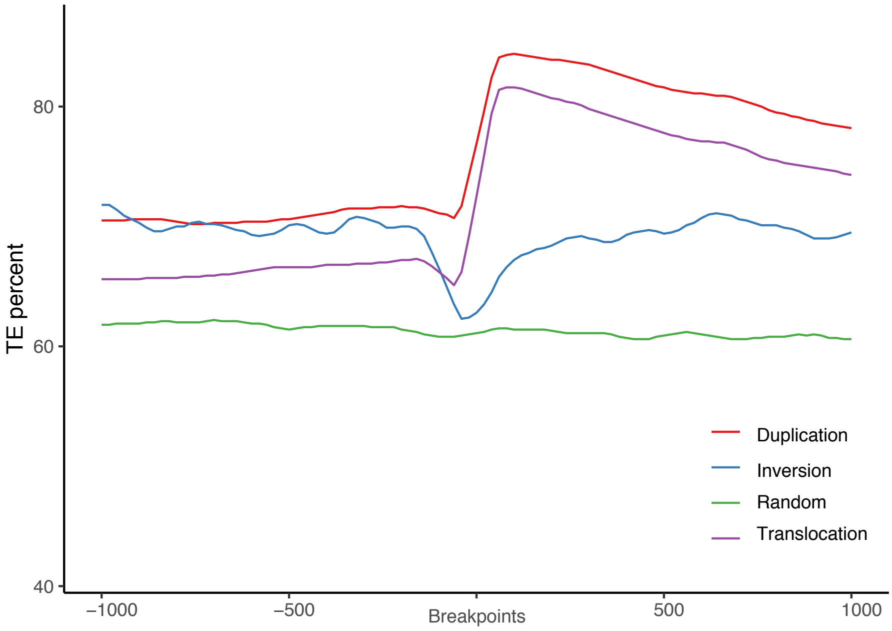

Supplementary Figure 6. Enrichment of transposon elements in structural variations. TE percent is much higher in duplications and translocations, but not in inversions.

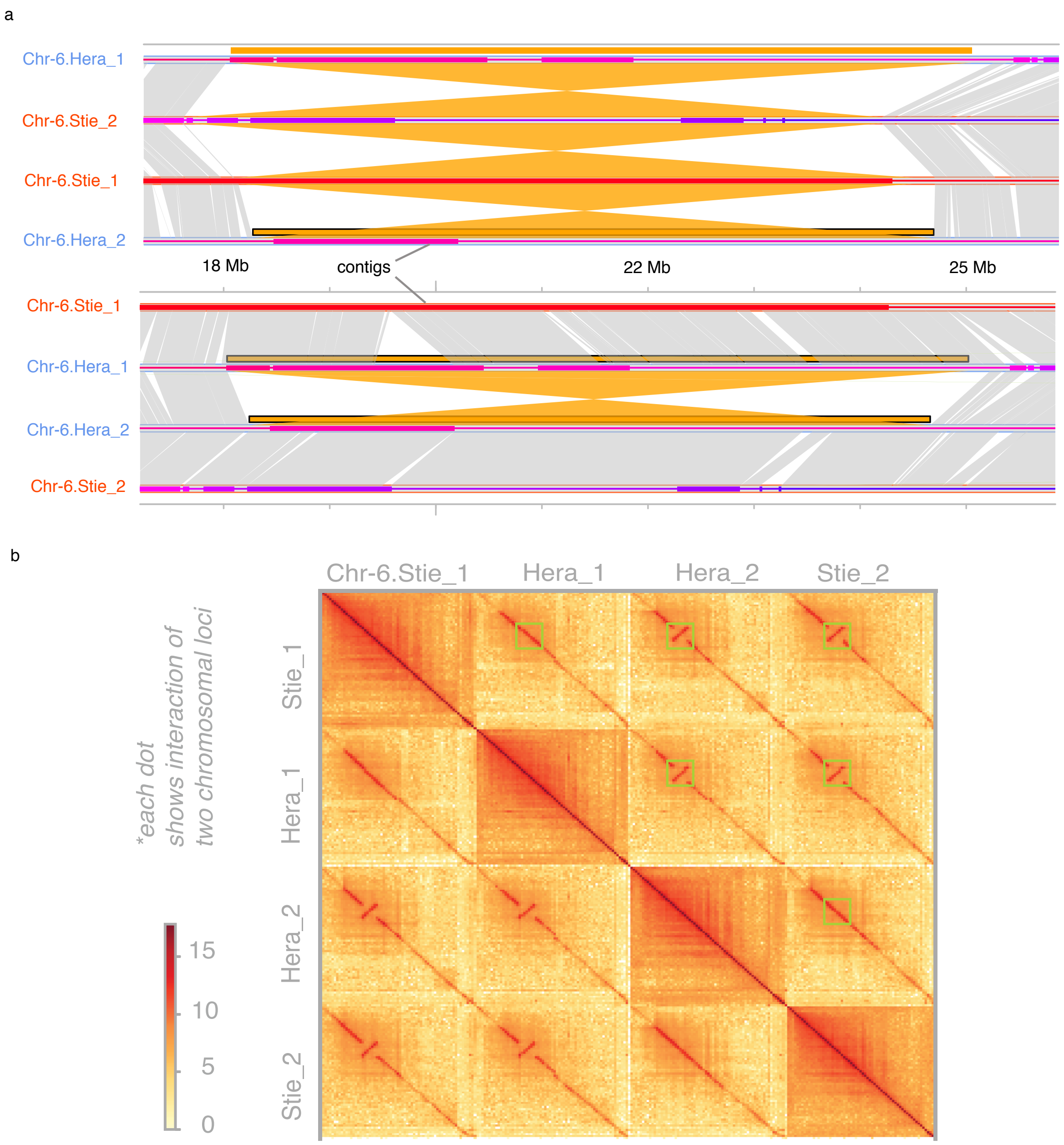

Supplementary Figure 7. Example for validation of structural variation. a: Pairwise comparison of the four haplotypes of Chr. 6 revealed four inversions:

Chr-6.Hera_1:18,032,256-25,020,440 versus *Chr-6.Stie_2:17,620,164-24,170,977*,
Chr-6.Stie_2:17,620,163-24,170,977 versus *Chr-6.Stie_1:18,104,687-24,467,329*,
Chr-6.Stie_1:18,104,687-24,467,329 versus *Chr-6.Hera_2:18,241,689-24,660,203*,
Chr-6.Hera_1:18,032,256-25,020,440 versus *Chr-6.Hera_2:18,241,690-24,660,203*.

Two other pairwise comparisons, i.e., *Chr-6.Stie_1* and *Chr-6.Hera_1, Chr-6.Hera_2* and *Chr-6.Stie_2*, showed high levels of synteny in the respective regions. All the regions involving the breakpoints of the inversions ended within contigs. **b**. The same inversions/synteny relationship between haplotypes were observed in Hi-C contact map. Together with information from **a**, these data evidenced that the inversions are real.

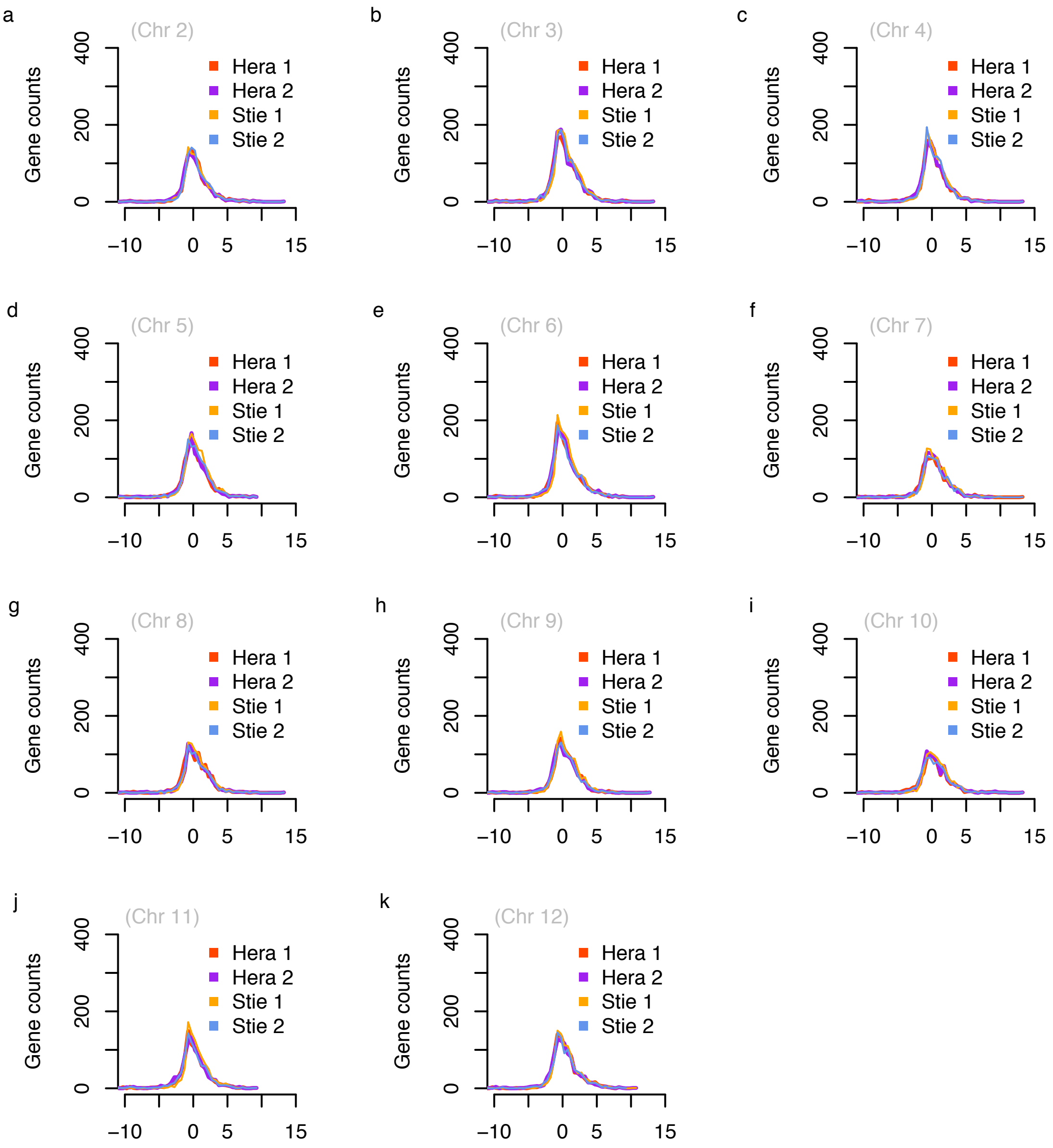

Supplementary Figure 8. Four haplotypes of chromosome 2-12 (respectively shown by panels a-k) showed comparable amount of gene expression (FPKM: fragments per kilobase per million reads). Note, chromosome 1 was given in main text Fig. 4d.

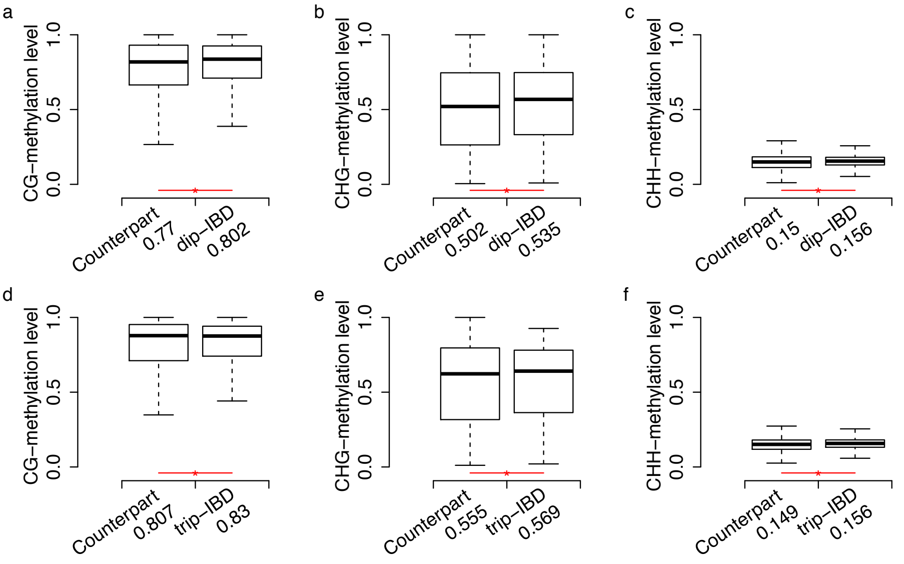

Supplementary Figure 9. Comparison of methylation (at CG, CHG, CHH context) between IBD blocks (of 50 kb) and their counterparts (i.e., genomic regions in synteny with related IBDs). a-c. Level of CG/CHG/CHH methylation related to IBD blocks shared by two haplotypes (labeled with “dip-IBD”). d-f. Level of CG/CHG/CHH methylation related to IBD blocks shared by three haplotypes (labeled with “trip-IBD”). Mean values of methylation levels (Materials and Methods) among the investigated blocks are given after *x*-axis labels. The number of analyzed dip-IBD blocks was 4,706, and that of dip-IBD counterpart blocks was also 4,706. Number of analyzed trip-IBD blocks was 2,925, and that of trip-IBD counterpart blocks was 975. The red line with an asterisk under each pair of boxes indicates that there is a significant difference between the two sets (two-sided *t*-test, *p*-values for a-f: 2.2e-16, 2.2e-16, 2.2e-16, 1.4e-09, 1.1e-02, 1.3e-11). In general, the methylation level at IBD blocks were slightly but significantly higher than that at their non-IBD counterparts. For example, in **a**, the average CG-methylation level of the 4,706 dip-IBD blocks was 0.802 and the methylation level of their counterparts was 0.77, while the former was siginicantly higher than the latter. Intervals for boxplots: center=median (50th percentile), lower bounds of box=25th percentile (Q1) and upper bound of box=75th percentile (Q3), lower whisker=maximum of (minima, Q1-1.5*IQR), upper whisker=minimum of (maxima, Q3+1.5*IQR). IQR=interquartile range (i.e., range of Q1 to Q3).

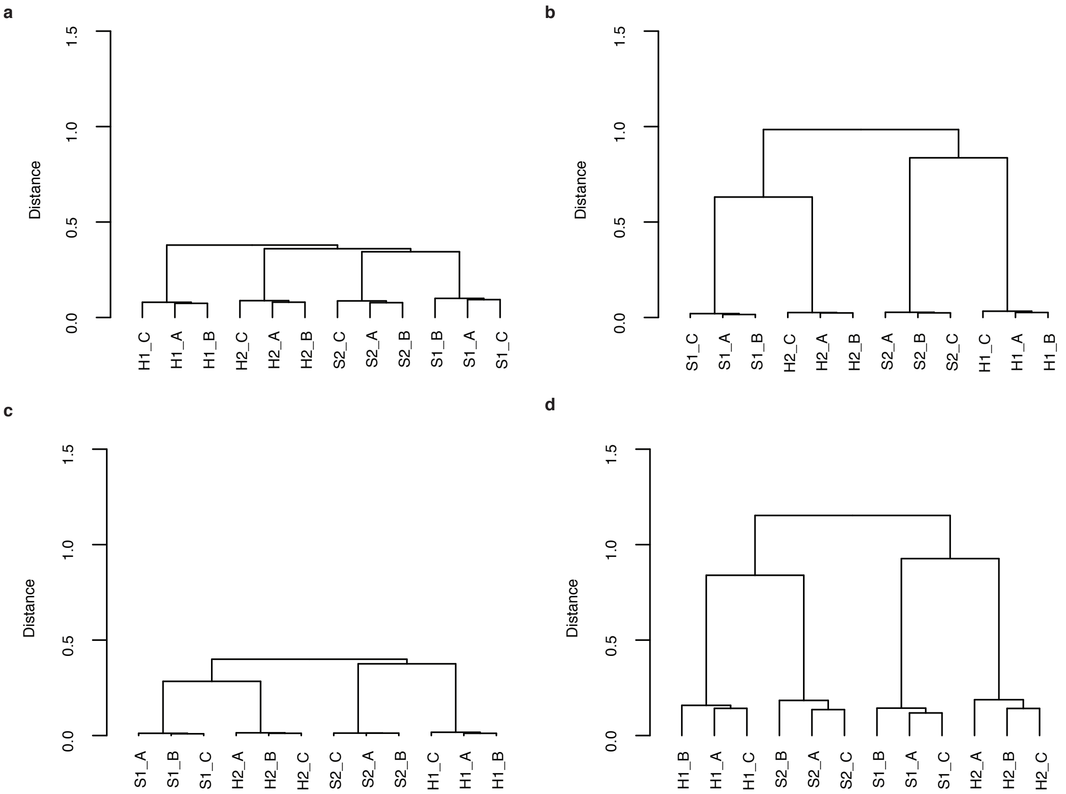

Supplementary Figure 10. High consistency between the biological replicates of sequencings of gene expression or DNA methylation as shown by well-clustered haplotypes. a. Clustering of replicates regarding haplotypes using haplotype-specific genome-wide allele expression. **b.** Clustering of replicates regarding haplotypes using haplotype-specific genome-wide methylation in CG context. **c.** Clustering of replicates regarding haplotypes using haplotype-specific genome-wide methylation in CHG context. **d.** Clustering of replicates regarding haplotypes using haplotype-specific genome-wide methylation in CHH context.

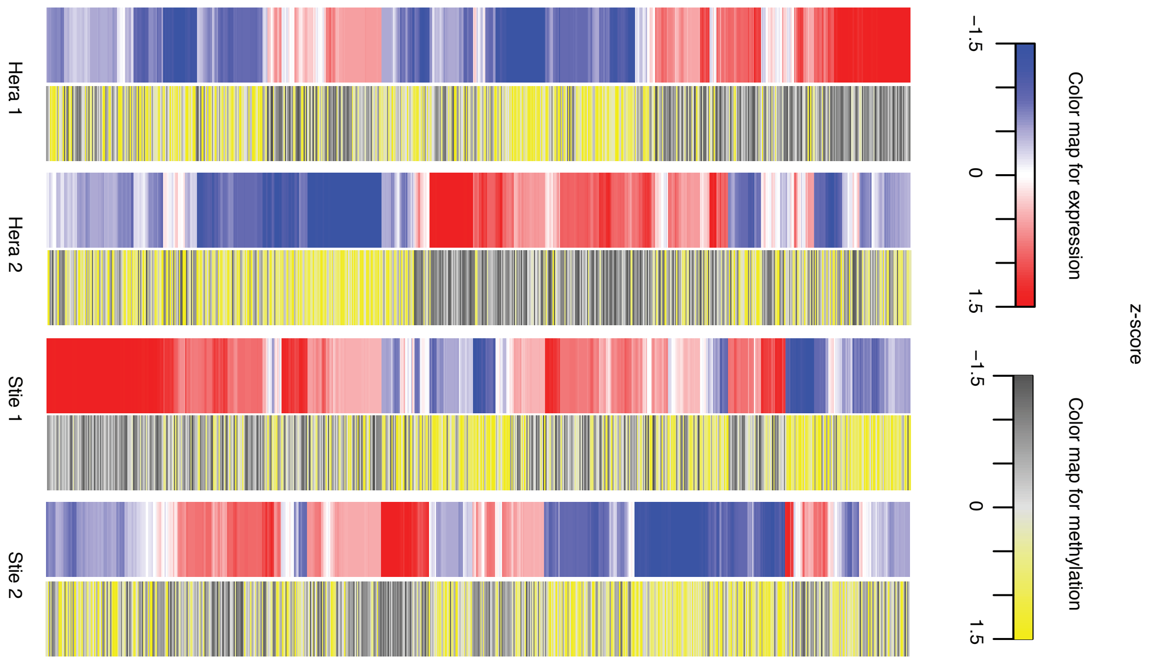

Supplementary Figure 11. Correlation of allele-specific expression (in blue/red) of 735 genes with DNA methylation level (in gray/yellow). Note, these genes showed negatively correlated relationship between expression and methylation which were measured in the 1 kb up- or downstream regions surrounding the genes, however, no significance was detected (two-sided correlation test, exact correlation values and *p*-values (>=0.05) provided in Supplementary Table 14). Combining with the 327 genes presented in main text Fig. 4g, over 87% of the differentially expressed genes showed negative correclation between expression and methylation. The remaining 157 genes that did not show a negative correlation between expression and methylation were not included here.

Supplementary Reference

1. Hutten, R.C.B. and van Berloo, R.. An online potato pedigree database. URL: http://www.plantbreeding.wur.nl/PotatoPedigree/ (2001).
2. van Berloo, R., Hutten, R.C.B., van Eck, H.J., and Visser, R.G.F. An online potato pedigree database resource. *Potato research* **50**, 45-57 (2007).
3. The Potato Genome Sequencing Consortium. Genome sequence and analysis of the tuber crop potato. *Nature* **475**, 189-195 (2011).
4. Pham, G.M., Hamilton, J.P. *et al*. Construction of a chromosome-scale long-read reference genome assembly for potato. *GigaScience* **9**, 1-11 (2020).
5. Quinlan, A.R., Hall, I.M.. *BEDTools*: A flexible suite of utilities for comparing genomic features. *Bioinformatics* **26**, 841-842 (2010).
6. Altschul, S.F., Gish, W., Miller, W., Myers, E.W. & Lipman, D.J.. Basic local alignment search tool. *J. Mol. Biol.* **215**, 403-410 (1990).
7. Rhie, A, Walenz, B.P., Koren, S., Phillippy, A.M.. *Merqury*: reference-free quality, completeness, and phasing assessment for genome assemblies. *Genome Biol* **21**, 1-27 (2020).
8. Wucher, V., Legeai, F., Hédan, B., Rizk, G., Lagoutte, L. et al. *FEELnc*: a tool for long non-coding RNA annotation and its application to the dog transcriptome. *Nucleic Acids Res.*, **45**, e57 (2017).
9. Kang, Y.J., Yang, D.C., Kong, L., Hou, M., Meng, Y.Q., Wei, L., Gao, G.. CPC2: a fast and accurate coding potential calculator based on sequence intrinsic features. *Nucleic Acids Res.* **45**, W12-W16 (2017).
10. UniProt Consortium. Activities at the Universal Protein Resource (UniProt). *Nucleic Acids Res.* **42**, D191-8 (2014).
11. Zhao, X., Li, J., Lian, B., Gu, H., Li, Y., Qi, Y.. Global identification of Arabidopsis lncRNAs reveals the regulation of MAF4 by a natural antisense RNA. *Nat Commun* **9**, 5056 (2018).
12. Emms, D.M., Kelly, S.. *OrthoFinder*: Phylogenetic orthology inference for comparative genomics. *Genome Biol.* **20**, 1-14 (2019).

13. Hu, X. *et al*. *pIRS*: Profile-based Illumina pair-end reads simulator. *Bioinformatics* **28**, 1533-1535 (2012).
