# Supplementary material for: Chromosome-scale and haplotype-resolved genome assembly of a tetraploid potato cultivar
Source: Nat Genet. 2022 Mar 3;54(3):342–8. doi: 10.1038/s41588-022-01015-0 (PMC8920897; doi:10.1038/s41588-022-01015-0)
Supplement: Supplementary file 2 — Reporting Summary [file 41588_2022_1015_MOESM2_ESM.pdf]

nature portfolio

Corresponding author(s): Korbinian Schneeberger
Last updated by author(s): Dec 21, 2021

Reporting Summary

Nature Portfolio wishes to improve the reproducibility of the work that we publish. This form provides structure for consistency and transparency in reporting. For further information on Nature Portfolio policies, see our Editorial Policies and the Editorial Policy Checklist.

Statistics

For all statistical analyses, confirm that the following items are present in the figure legend, table legend, main text, or Methods section.

n/a	Confirmed	
☐	☒	The exact sample size (*n*) for each experimental group/condition, given as a discrete number and unit of measurement
☒	☐	A statement on whether measurements were taken from distinct samples or whether the same sample was measured repeatedly
☐	☒	The statistical test(s) used AND whether they are one- or two-sided<br>*Only common tests should be described solely by name; describe more complex techniques in the Methods section.*
☒	☐	A description of all covariates tested
☐	☒	A description of any assumptions or corrections, such as tests of normality and adjustment for multiple comparisons
☐	☒	A full description of the statistical parameters including central tendency (e.g. means) or other basic estimates (e.g. regression coefficient) AND variation (e.g. standard deviation) or associated estimates of uncertainty (e.g. confidence intervals)
☐	☒	For null hypothesis testing, the test statistic (e.g. *F*, *t*, *r*) with confidence intervals, effect sizes, degrees of freedom and *P* value noted<br>*Give P values as exact values whenever suitable.*
☒	☐	For Bayesian analysis, information on the choice of priors and Markov chain Monte Carlo settings
☒	☐	For hierarchical and complex designs, identification of the appropriate level for tests and full reporting of outcomes
☒	☐	Estimates of effect sizes (e.g. Cohen's *d*, Pearson's *r*), indicating how they were calculated

Our web collection on statistics for biologists contains articles on many of the points above.

Software and code

Policy information about availability of computer code

Data collection	No code used for data collection.
Data analysis	All routine analysis were performed using tools publicly available.<br>Barcodes were corrected using cellranger (10x Genomics, v1.1.0).<br>Read quality control: Trimmomatic (v0.39).<br>Short/long reads were aligned using bowtie2 (2.2.8)/minimap2 (2.20-r1061), hisat2 (v2.2.0).<br>BAM, VCF file processing and sequencing depth analysis were performed using samtools (v1.9) and bedtools (v2.27.1).<br>PacBio sequence reads were assembled using hifiasm (v0.7).<br>Contig polishing: pilon (v1.22) and racon (1.4.10).<br>Structural variations were identified using SyRI (v1.0) based on minimap2 genome alignments.<br>Methylation calling was performed with bismark (v0.23.0) pipeline.<br>Genome size was estimated using findGSE (v1.0).<br>k-mers were counted and handled using jellyfish (v2.2.10), kmc3 (v3.1.1) and Merqury (v1.3).<br>Statistical analysing including t-test and correlation test were performed using R (v3.5.1).<br>Blast of nucleotides was performed with blast tool kit (v2.10.0+).<br>Hi-C data was processed using ALLHiC (v0.9.8) and SALSA2 (v2.2) pipeline.<br>BUSCO analysis using BUSCO (version 4.1.4).<br>Genome annotation: Augustus (version 3.2.3), GlimmerHMM (version 3.0.1), SNAP (v2006-07-28), exonerate (v2.2.0), StringTie (v1.3.4d), EvidenceModeler (version not available), RepeatModeler (v2.0.1), RepeatMasker (open-4.0), Infernal (v1.1).<br>RNA-seq quantification: HTSeq (v0.13.5).<br>Replicates of expression and methylation clustering: package hclust (v3.5.1) in R.<br>Protein sequence alignment: blastp (v2.10.0+) and Scipio (v1.4.1).<br>Functional annotation: InterProscan (v5.48) and R package ClusterProfiler (v3.14).

Protein clustering: OrthoFinder (v2.2.6).
Transcript checking: FEELnc (v.0.1.1) and CPC2 (v0.9-r2).
Custome code and scripts supporting this work is available at github.com/schneeberger-lab/GameteBinning_tetraploid or Zenodo under Creative Commons Attribution 4.0 International license (DOI: 10.5281/zenodo.5775114).

For manuscripts utilizing custom algorithms or software that are central to the research but not yet described in published literature, software must be made available to editors and reviewers. We strongly encourage code deposition in a community repository (e.g. GitHub). See the Nature Portfolio guidelines for submitting code & software for further information.

Data

Policy information about availability of data

All manuscripts must include a data availability statement. This statement should provide the following information, where applicable:

- Accession codes, unique identifiers, or web links for publicly available datasets
- A description of any restrictions on data availability
- For clinical datasets or third party data, please ensure that the statement adheres to our policy

High-throughput sequencing data analyzed in this project are available under NCBI BioProject: PRJNA751899. This Whole Genome Shotgun project (including assembly and annotation) has been deposited at DDBJ/ENA/GenBank under the accessions JAIVGA000000000, JAIVGB000000000, JAIVGC000000000 and JAIVGD000000000. The version described in this paper is version JAIVGA010000000, JAIVGB010000000, JAIVGC010000000 and JAIVGD010000000. The genome assembly and gene annotation of 'Otava' are also available on Spud DB (http://spuddb.uga.edu/)6. Source data are provided at Zenodo under Creative Commons Attribution 4.0 International license (DOI: 10.5281/zenodo.5796752). Genome information of DM and RH used in this study are available on Spud DB (http://spuddb.uga.edu/).

All the databases/datasets used in the study are along with appropriately accessible links/accession-codes in the manuscript under the "Data availability" section as well as in this reporting summary.

Field-specific reporting

Please select the one below that is the best fit for your research. If you are not sure, read the appropriate sections before making your selection.

☒ Life sciences ☐ Behavioural & social sciences ☐ Ecological, evolutionary & environmental sciences

For a reference copy of the document with all sections, see nature.com/documents/nr-reporting-summary-flat.pdf

Life sciences study design

All studies must disclose on these points even when the disclosure is negative.

Sample size	Three samples/cultivars were selected, including Hera, Stieglitz and Otava, which make a trio for genome and haplotyping analysis.
Data exclusions	No data were excluded from the analysis.
Replication	Three biological replicates were respectively used for both RNA and methylation sequencing and analysis. All attempts at replication were successful.
Randomization	This is not relevant to this study, as it is about assembly and analysis of a single cultivar.
Blinding	No group allocation was needed in this study.

Reporting for specific materials, systems and methods

We require information from authors about some types of materials, experimental systems and methods used in many studies. Here, indicate whether each material, system or method listed is relevant to your study. If you are not sure if a list item applies to your research, read the appropriate section before selecting a response.

Materials & experimental systems

n/a	Involved in the study	
☒	☐	Antibodies
☒	☐	Eukaryotic cell lines
☒	☐	Palaeontology and archaeology
☒	☐	Animals and other organisms
☒	☐	Human research participants
☒	☐	Clinical data
☒	☐	Dual use research of concern

Methods

n/a	Involved in the study	
☒	☐	ChIP-seq
☒	☐	Flow cytometry
☒	☐	MRI-based neuroimaging
